# Progress of the application clinical prediction model in polycystic ovary syndrome

**DOI:** 10.1186/s13048-023-01310-2

**Published:** 2023-11-25

**Authors:** Guan Guixue, Pu Yifu, Gao Yuan, Liu Xialei, Shi Fan, Sun Qian, Xu Jinjin, Zhang Linna, Zhang Xiaozuo, Feng Wen, Yang Wen

**Affiliations:** 1 The First People’s Hospital of Lianyungang, Lianyungang, Jiangsu 222002 China; 2 Xuzhou Medical University affiliated hospital of Lianyungang, Lianyungang, Jiangsu 222002 China; 3 The first affiliated hospital of Kangda College of Nanjing Medical University, Lianyungang, Jiangsu 222002 China; 4 Laboratory of Genetic Disease and Perinatal Medicine, Key laboratory of Birth Defects and Related Diseases of Women and Children, Ministry of Education, West China Second University Hospital, Sichuan University, Chengdu, Sichuan 610041 China

**Keywords:** Clinical prediction model, Polycystic ovary syndrome, Application progress, Reproduction, Endocrine, Overweight PCOS

## Abstract

Clinical prediction models play an important role in the field of medicine. These can help predict the probability of an individual suffering from disease, complications, and treatment outcomes by applying specific methodologies. Polycystic ovary syndrome (PCOS) is a common disease with a high incidence rate, huge heterogeneity, short- and long-term complications, and complex treatments. In this systematic review study, we reviewed the progress of clinical prediction models in PCOS patients, including diagnosis and prediction models for PCOS complications and treatment outcomes. We aimed to provide ideas for medical researchers and clues for the management of PCOS. In the future, models with poor accuracy can be greatly improved by adding well-known parameters and validations, which will further expand our understanding of PCOS in terms of precision medicine. By developing a series of predictive models, we can make the definition of PCOS more accurate, which can improve the diagnosis of PCOS and reduce the likelihood of false positives and false negatives. It will also help discover complications earlier and treatment outcomes being known earlier, which can result in better outcomes for women with PCOS.

**Supplementary Information:**

The online version contains supplementary material available at 10.1186/s13048-023-01310-2.

## Background

Clinical prediction models use specific methodologies to predict individual probabilities of illness, complications, treatment outcomes, and includes a diagnostic model and a prognostic model [1]. There are several basic steps from model creation to model application. It includes the formulation of research questions, assessment design, data collection, establishment and evaluation of clinical prediction models, validation and application, and updating of models [2]. Researchers have created a tool called PROBAST to assess the risk of bias and applicability of a prediction model. It is also used in a specific population and has shown initial success [3–6]. Clinical prediction models use a small number of predictors to predict disease status and prognosis. These predictors can be easily collected and detected.

Recently, several emerging technologies have been used in clinical prediction models. For example, computer technology, interdisciplinary communication, application of big data, and many advanced methods such as machine learning and artificial intelligence were applied. Combining these new technologies with a clinical prediction model can ensure proper allocation of medical resources. Moreover, it can achieve the goals of the three levels of prevention.

Clinical prediction models have been widely used in the medical field, including prediction models for tumor diseases [7–9], non-tumor diseases [10–12], and obstetrics and gynecology diseases [13–15]. In recent years, clinical prediction models have been extensively used in the field of reproduction. For example, the application of clinical prediction models for polycystic ovary syndrome (PCOS) has been increasingly studied. PCOS is one of the most common reproductive disorders. It has attracted the most attention because of its high incidence, considerable heterogeneity, and complex treatments. Moreover, it can also cause short- and long-term complications that require long-term management [16]. Several studies have focused on PCOS by creating models from different perspectives.

In this study, we reviewed and interpreted the progress of clinical prediction models for PCOS from different perspectives. With the help of this study, we provided a comprehensive review of related research and a better guide for clinical management. Moreover, it can achieve more accurate treatment for individual patients with PCOS.

## Methods

We conducted a comprehensive literature systematic review up to April 2023, utilizing PubMed, Embase, and Web of Science databases. Our search terms encompassed “polycystic ovary syndrome” or “PCOS” along with “prediction model,” “nomogram,” or “model.” We limited our selection to research papers published in English and prioritized those published since 2020, excluding any lacking full manuscripts.

## Diagnosis standards and guidelines or recommendations of PCOS

PCOS is the most common endocrine disorder in women of reproductive age, affecting 6–10% of women of reproductive age [17]. There are many guidelines and diagnostic standards for PCOS, including the National Institutes of Health (NIH) standard in 1990 [18], Rotterdam Standard in 2003 [19], Androgen Excess Society (AES) standard in 2006 [20], PCOS Diagnosis and Treatment Guidelines issued by the Endocrine Society of America in 2012 [21], and recommendations from international evidence-based guidelines for the assessment and management of polycystic ovary syndrome in 2018 [22]. The most widely used standard for PCOS is the Rotterdam 2003 criteria. PCOS is defined as sporadic menstruation, sporadic ovulation or non-ovulation, blood biochemistry or clinical signs of hyperandrogenemia except from other diseases, and ultrasound suggesting polycystic changes, conforming to two out of three contents [19].

## Application of clinical prediction model in PCOS

**Fig. 1:**
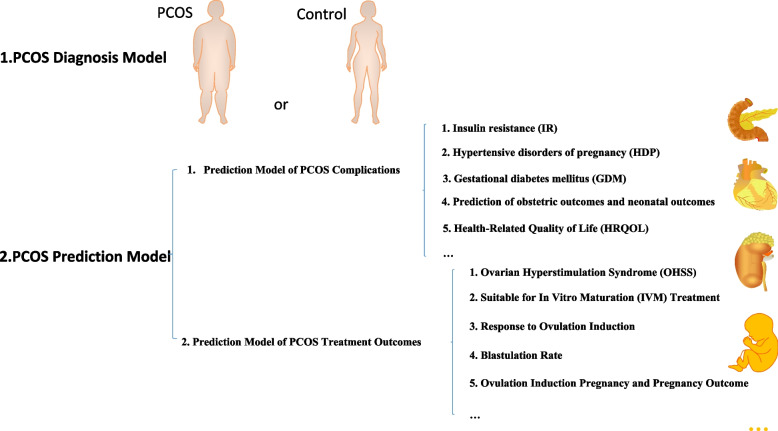
A summary of progress of the application clinical prediction model in polycystic ovary syndrome

As shown in Fig. 1, we have primarily categorized the prediction models for PCOS into two types, encompassing diagnostic models for PCOS, prediction models for PCOS (mainly divided into two categories: Prediction Model of PCOS Complications and Prediction Model of PCOS Treatment Outcomes).

### PCOS diagnosis model

Studies have shown that up to 75% of PCOS cases have not been identified in clinical practice, which inevitably leads to missed diagnoses [23]. Identifying individuals at high risk of PCOS in precision medicine could help achieve early treatments.

In 2007, Pedersen et al. used questionnaires to diagnose PCOS based on demographic information, medical history, related disease diagnosis, menstrual history, reproductive history, and other relevant factors. It can be useful for screening women with menstrual irregularities, hirsutism, or other related findings consistent with PCOS. However, the questionnaire has not been validated in a family medicine setting. A score ≥ 2 was deemed as PCOS [24]. Zhang et al. used a supervised machine learning algorithm to predict PCOS genes by comparing 306 PCOS genes as positive samples and 306 negative samples from 13,681genes. In total, 233 PCOS genes were identified in this study. This opens a new avenue for identifying patients at risk of PCOS [25]. Similarly, Deshmukh et al. identified four independent predictors of PCOS, including free androgen index (FAI), anti-Müllerian hormone (AMH), waist circumference (WC), and 17OHP (17α-hydroxyprogesterone). However, there are certain limitations, including the lack of external validation and inability to evaluate the other three PCOS phenotypes [26]. Sun et al. found that several coagulation parameters (prothrombin time, thrombin time, and fibrin degradation products) were predictive of PCOS. These results highlighted the potential of anticoagulation therapies to lower the risk of adverse outcomes in PCOS patients [27].

Notably, by using polygenic risk prediction, Joo YY et al. identified phenome-wide comorbidity patterns characteristic of PCOS that improved diagnostic accuracy [23]. Two initial genome-wide association studies (GWAS) identified several new susceptibility loci for PCOS. Both GWAS were performed in Han Chinese populations [28, 29]. These new susceptibility loci were subsequently replicated in European cohorts [30–33]. A large-scale genome-wide meta-analysis of PCOS suggested a shared genetic architecture for the different diagnostic criteria [34]. The above studies have improved our ability to diagnose and treat PCOS.

Vagios et al. constructed an age-adjusted model that combined AMH and Body Mass Index (BMI) to predict oligo-anovulation diagnosis (mainly PCOS). This model served as a tool for patient counselling in the setting of anovulatory infertility [35]. Strowitzki et al. appraised this prediction model and pointed out that it can be used as a good tool, especially in patients with a minor clinical form of PCOS [36].

Importantly, the prevalence of PCOS in adolescents increases by up to 30% [37]. Li M et al. found that combining AMH and testosterone (TT) may be used in a multivariate predictive model to predict PCOS in adolescent girls [38].

With the COVID-19 global pandemic in mind, Zigarelli et al. developed two types of models using machine learning techniques. The models can help users acquire pre- or self-diagnosis and counsel for the risk of PCOS with or without lab tests and access the platform at home without delay [39]. The details of the above studies are provided in Supplement Material 1.

### PCOS prediction model

#### Prediction model of PCOS complications

PCOS is always accompanied by obesity, metabolic dysfunction, short-term complications, and long-term complications. It is associated with an increased risk of delivery complications during pregnancy [40, 41].

#### Insulin resistance (IR)

IR is an underlying feature of PCOS. Up to 75% of PCOS patients have IR [42]. It can be measured, but there is no tool to predict whether patients will develop IR. Hence, in 2020, Jiang et al. developed a prediction model to evaluate an individual PCOS patient’s risk of IR. The model includes five predictors: occupational category, disease duration (years), BMI, metformin use, and physical activity [43]. Previous studies have explored the factors influencing insulin sensitivity in PCOS patients and found that sex hormone-binding globulin (SHBG) and other indicators can predict IR. However, there are certain limitations, mainly including small sample size and lack of external verification [44, 45].

#### Hypertensive disorders of pregnancy (HDP)

In 2021, Khomami et al. [46] performed the Australian Longitudinal Study on Women’s Health and clarified the impact of PCOS on the incidence of HDP. The key control variables included in this study were age, BMI, country of birth, parity, multiple pregnancies, infertility treatment, gestational diabetes mellitus (GDM), family history of GDM, and socioeconomic status. Univariate analysis showed that PCOS positively correlated with the incidence of HDP. Further subgroup analysis showed that PCOS was also positively associated with HDP incidence in nonobese women. A meta-analysis and population-based study on 9.1 million pregnancies proved that PCOS is an independent risk factor for HDP [47, 48].

##### GDM

Pregnant women with PCOS have an increased likelihood of developing GDM compared with normal pregnant women [49]. A study involving 326 PCOS patients (148 PCOS patients without GDM and 41 PCOS patients with GDM) used certain factors to develop a clinically useful prediction model to predict the risk of GDM in PCOS patients [50]. Risk factors include type 2 diabetes in a first-degree relative, fasting glucose, fasting insulin, androstenedione, and SHBG. It is noteworthy that the model was not externally validated. Therefore, this model should be used with caution. In addition, Wang et al. developed a simple model to predict the risk of GDM in the first trimester without using blood examination indexes. Predictors including pre-pregnancy BMI, abdominal circumference in the first trimester, age, PCOS, gravidity, irregular menstruation, and family history of diabetes were easily obtained in the first trimester at the primary health care center. The model can be easily used and would facilitate intervention plans for maternal and infant healthcare to prevent the risk of GDM in early pregnancy [51].

##### Prediction of obstetric outcomes and neonatal outcomes

Studies have shown that women with PCOS have worse obstetric and neonatal outcomes than normal females [52–54]. A review pointed out that PCOS patients had an increased risk of pregnancy complications and adverse child outcomes in that PCOS is associated with heterogeneous etiological factors and co-morbidities [55]. In 2020, Christ et al. identified the association between baseline characteristics of PCOS patients and major obstetric and perinatal complications. The results suggested that the primary disease characteristics of PCOS, chiefly hyperandrogenism and changes in blood glucose regulation, are relevant in predicting obstetric complications among women with PCOS [56]. Therefore, pre-pregnancy intervention may be a good way to reduce the risk of adverse pregnancy outcomes in patients with PCOS.

##### Health-related quality of life (HRQOL)

To explore the factors that contribute to HRQOL and improve or maintain HRQOL in PCOS patients, Bazarganipour et al. found that the most significant predictors of HRQOL in patients with PCOS include self-esteem, body image, and sexual function. They recommended that healthcare providers should be made aware of HRQOL impairment in women with PCOS, which requires appropriate management and adequate treatment if available [57, 58].

Details of the above studies are provided in Supplement Material 2.

#### Prediction model of PCOS treatment outcomes

##### Ovarian Hyperstimulation syndrome (OHSS)

Controlled ovarian hyperstimulation (COH) may be one treatment option for some PCOS patients who are experiencing infertility and have difficulty ovulating, it is not a universal requirement for all PCOS patients. This process of COH is often difficult to control and can result in multiple follicle development, multiple pregnancies, and OHSS [59, 60]. OHSS is a common iatrogenic complication that occurs after ovulation hyperstimulation and is accompanied by pleural and abdominal effusions, dyspnea, blood hypercoagulability, and in severe cases, death [61].

Li et al. in 2021 investigated the risk factors of OHSS in PCOS patients after in vitro fertilization/intracytoplasmic sperm injection (IVF/ICSI). They developed an equation describing the probability of OHSS:

$$\text{P}=-3.47315\;\text{to}\;0.05521\times\text{FSH}+0.24700\times\text{AMH}+0.00014\times\text{total}\;\text{dosage}\;\text{of}\;\text{Gn}\;\text{used}+0.00015\times\text{E}2\;\text{value}\;\text{on}\;\text{the}\;\text{day}\;\text{of}\;\text{hCG}\;\text{injection}+0.07249\times\text{follicle}\;\text{number}\;\text{on}\;\text{the}\;\text{day}\;\text{of}\;\text{hCG}\;\text{injection}$$

This is a simple, intuitive, practicable, and valuable method for clinical applications [62]. Another study in 2021 [63] explored the effect of seasonality on high-risk OHSS patients after oocyte retrieval. A total of 2030 patients with PCOS infertility underwent the follicular phase protocol. The risk of OHSS is seasonal and more serious during the summer and winter. Gn dosage, number of oocytes collected, E2 values, average diameter of both ovaries on hCG injection day, type of infertility, bilateral average ovarian diameter on hCG injection day, infertility type, and average temperature were independent risk factors. They found that the incidence of OHSS features important seasonal fluctuations; moreover, extreme weather conditions increase the risk of OHSS.

##### Suitable for in vitro maturation (IVM) treatment

Currently, there is some controversy regarding whether PCOS patients should undergo IVF or IVM. A cohort study that included 124 PCOS patients who received continuous IVM treatment found that in this population, AMH and AFC are good predictor factors to guide the patient in choosing IVM treatment or obtain at least eight cumulus oocyte complexes (COC) available for IVM culture [64]. The study addressing the indications for IVM will surely provide a relatively accurate screening standard.

##### Response to ovulation induction

Some prediction models have been developed for this purpose in women with WHO type II anovulation, which encompasses a subgroup of PCOS patients [65–72]. The predictive variables identified in these studies were age, duration of infertility, BMI, measures of hyperandrogenism, and measures of insulin resistance because 91% of women with WHO type II anovulation are diagnosed with PCOS according to the Rotterdam criteria [73].

Importantly, Verit et al. found that total antioxidant capacity (TAC), ovarian volume (OV), and FAI could predict the response to ovulation induction to clomiphene citrate (CC) in nonobese PCOS patients. However, considering the small sample size and large heterogeneity of PCOS, these conclusions require further verification [74]. In addition, van Wely et al., by assessing the external validity of the model predicting the individual FSH response dose, found that it was inadequate in CC-resistant PCOS patients undergoing ovulation induction with recombinant FSH (rFSH) in a low-dose step-up regimen [75].

##### Blastulation rate

Jin et al. developed a nomogram to predict the probability of extended culture to the blastocyst stage in women undergoing IVF for PCOS. The model exhibited fair performance and was well calibrated. If these predictive models are confirmed by external validation and the sample size is increased, they will represent convenient and practical tools to help guide the management of patients about extending culture to the blastocyst stage [76].

##### Ovulation induction pregnancy and pregnancy outcome

PCOS patients often suffer from oligo-ovulation or anovulation. Therefore, a prediction model for ovulation induction and pregnancy-related outcomes is particularly important.

In 2015, Kuang et al. performed a secondary analysis of previously reported data from PCOS-I [70] and II trials [77]. The PCOS-I and PCOS-II trials included 626 and 750 patients, respectively. In the PCOS-I experiment, participants were randomly allocated to receive CC, metformin, or a combination of CC and metformin. In the PCOS-II experiment, participants were randomly allocated to receive either letrozole or CC. The investigators assessed the efficiency of the previous models and constructed a new predictive model based on the method of treatment, BMI, and other published variables. They found that the predictive factors were similar between PCOS-I and PCOS-II. Age, FAI, insulin, time of conception, and SHBG level were important predictors of pregnancy outcomes. Moreover, they revealed that maternal smoking during pregnancy was associated with adverse pregnancy outcomes [78].

Based on clinical, ultrasonographic, and endocrinological parameters, van Wely et al. predicted ongoing pregnancy following ovulation induction with rFSH in women with PCOS. They also constructed a model that can be used in counselling of women with CC-resistant PCOS [69]. Gao et al. developed a nomogram and formula for live birth:

$$P=\frac{1}{\left[1+\exp (2X)\right]}$$

Where X is:

$$X=0.50654-0.57801\times \textrm{V}1-0.20514\times \textrm{V}2-0.08219\times \textrm{V}3+0.43403\times \textrm{V}4+0.90164\times \textrm{V}5$$

V1 is the type of embryo transferred, V2 is total serum cholesterol (TC), V3 is basal FSH serum level, V4 is BMI, and V5 is age. It was developed to predict the live birth rate in PCOS patients [79]. Similarly, Jiang et al. predicted live births in PCOS patients who were undergoing frozen-thawed embryo transfer (FET) and constructed a nomogram model capable of predicting live birth outcomes [80]. Veltman-Verhulst et al. confirmed the overall good live birth prognosis of anovulatory women with PCOS who received conventional ovulation induction treatment according to age, infertility duration, and BMI. The clinical advantage of the identification of patients with poor prognosis allowed them to embark on alternative treatment options earlier [81]. Guan et al. predicted pregnancy after intrauterine insemination in women with PCOS and found that BMI plays a role in pregnancy outcomes, especially in obese women who require higher Gn doses and more days of COS to overcome the effects of weight [82]. The details of the above studies are provided in Supplement 3.

## Discussion

This review summarizes the current PCOS predictive models that have been reported, including the PCOS diagnosis model and predictive models (complications and treatment outcome models). PCOS is a heterogeneous disease that can be influenced by a variety of factors including genetic factors, environmental factors, and endocrine status [83]. The definition of PCOS evolved as knowledge of the disease continued to deepen. Overall, the definition of PCOS has become clearer [84, 85]. Hence, based on this review, we propose future research directions for PCOS predictive models. We live in an era of precision medicine in which PCOS incidence differs among different races, so it is necessary to make related prediction models based on different racial groups [86]. We can optimize the past prediction models and take advantage of the specificity and sensitivity to distinguish PCOS and other related diseases. There have been a handful of studies reporting that PCOS may frequently be concurrent with nonalcoholic fatty liver disease (NAFLD) [87, 88], obstructive sleep apnea syndrome [89, 90], diabetes [91, 92] and the like. Thus, we can also develop models predicting the occurrence of other related diseases in PCOS patients. Additionally, many studies on the relationship between gene polymorphism and PCOS have been reported [93–95]. We can predict PCOS by adding a genetic related predictor. Following up on the offspring of women with PCOS can help predict how to maximize the benefits in PCOS patients from the perspective of the offspring [96–98]. It has been reported that PCOS patients are prone to endometrial hyperplasia and endometrial cancer (EC) [99]. Moreover, PCOS may impair endometrial receptivity, which can increase adverse pregnancy outcomes [100, 101]. We can predict PCOS pregnancy outcomes by adding endometrial lesions predictor. By developing a series of predictive models, we can make the definition of PCOS more accurate, which can improve the diagnosis of PCOS and reduce the likelihood of false positives and false negatives. It will also help discover complications earlier and treatment outcomes being known earlier, which can result in better outcomes for women with PCOS.

## Conclusion

This study is the first review to summarize the PCOS prediction model, aiming to make more people aware of its wide application. It may enable doctors to counsel patients regarding the most effective and patient-tailored treatment strategy before treatment initiation. Notably, even if the models currently have poor prediction performance by internal and external validation, this does not mean that they are worthless [102]. As we all known, a model that performs well in an internal validation always produce poor predictions in other patients [103]. Hence, in the future, models with poor accuracy can be improved by adding well-known parameters and validations, which will further expand our understanding of this disease in terms of precision medicine.

### Supplementary Information

**Additional file 1:** **Supplement Material 1.** PCOS diagnosis model.

**Additional file 2:** **Supplement Material 2.** Prediction model of PCOS complications.

**Additional file 3:** **Supplement Material 3.** Prediction model of PCOS treatment outcomes.

## Data Availability

All data generated or analysed during this study are included in this article.

## Abbreviations

PCOS: polycystic ovary syndrome
NIH: National Institutes of Health
AES: Androgen Excess Society
AMH: anti-Müllerian hormone
ICSI: intracytoplasmic sperm injection
FET: frozen-thawed embryo transfer
NAFLD: nonalcoholic fatty liver disease
OV: ovarian volume
CC: clomiphene citrate
EC: endometrial cancer
TC: total serum cholesterol
SHBG: sex hormone-binding globulin
rFSH: recombinant FSH
FAI: free androgen index
WC: waist circumference
TAC: total antioxidant capacity
17OHP: 17α-hydroxyprogesterone
GWAS: genome-wide association studies
TT: testosterone
IVF: in vitro fertilization
IVM: in vitro maturation
COC: cumulus oocyte complexes
BMI: Body Mass Index
IR: Insulin resistance
HDP: Hypertensive disorders of pregnancy
GDM: gestational diabetes mellitus
HRQOL: Health-Related Quality of Life
OHSS: Ovarian Hyperstimulation Syndrome
COH: controlled ovarian hyperstimulation

## References

[1] Adams ST, Leveson SH (2012). Clinical prediction rules. BMJ.

[2] Ranstam J, Cook JA, Collins GS (2016). Clinical prediction models. Br J Surg.

[3] Hemingway H, Croft P, Perel P (2013). Prognosis research strategy [PROGRESS] 1: a framework for researching clinical outcomes. BMJ..

[4] Riley RD, Hayden JA, Steyerberg EW (2013). Prognosis research strategy [PROGRESS] 2: prognostic factor research. PLoS Med.

[5] Steyerberg EW, Moons KG, van der Windt DA (2013). Prognosis research strategy [PROGRESS] 3: prognostic model research. PLoS Med.

[6] Hingorani AD, Windt DA, Riley RD (2013). Prognosis research strategy [PROGRESS] 4: stratified medicine research. BMJ.

[7] Chen Q, Hu L, Chen K (2020). Construction of a nomogram based on a hypoxia-related lncRNA signature to improve the prediction of gastric Cancer prognosis. Front Genet.

[8] Sun Y, Li Y, Wu J (2020). Nomograms for prediction of overall and cancer-specific survival in young breast cancer. Breast Cancer Res Treat.

[9] Zheng Z, Zhou X, Zhang J (2021). Nomograms predict survival of patients with small bowel adenocarcinoma: a SEER-based study. Int J Clin Oncol.

[10] Dong YM, Sun J, Li YX (2021). Development and validation of a nomogram for assessing survival in patients with COVID-19 pneumonia. Clin Infect Dis.

[11] Dong W, Wan EYF, Fong DYT (2021). Prediction models and nomograms for 10-year risk of end-stage renal disease in Chinese type 2 diabetes mellitus patients in primary care. Diabetes Obes Metab.

[12] Wu Y, Hu H, Cai J (2020). A prediction nomogram for the 3-year risk of incident diabetes among Chinese adults. Sci Rep.

[13] Zheng T, Ye W, Wang X (2019). A simple model to predict risk of gestational diabetes mellitus from 8 to 20 weeks of gestation in Chinese women. BMC Pregnancy Childbirth.

[14] Chen Q, Wang S, Lang JH (2020). Development and validation of nomograms for predicting overall survival and Cancer-specific survival in patients with ovarian clear cell carcinoma. J Ovarian Res.

[15] Qi X, Fu Y, Zhang M (2020). An innovative immune score-based prognostic nomogram for patients with cervical Cancer. Biomed Res Int.

[16] Ruan X, Li M, Mueck AO (2018). Why does polycystic ovary syndrome [PCOS] need long-term management?. Curr Pharm Des.

[17] Yildiz BO, Bozdag G, Yapici Z, Esinler I, Yarali H (2012). Prevalence, phenotype and cardiometabolic risk of polycystic ovary syndrome under different diagnostic criteria. Hum Reprod.

[18] Legro RS (2003). Diagnostic criteria in polycystic ovary syndrome. Semin Reprod Med.

[19] Rotterdam EA-SPCWG (2004). Revised 2003 consensus on diagnostic criteria and long-term health risks related to polycystic ovary syndrome. Fertil Steril.

[20] Azziz R, Carmina E, Dewailly D (2006). Positions statement: criteria for defining polycystic ovary syndrome as a predominantly hyperandrogenic syndrome: an androgen excess society guideline. J Clin Endocrinol Metab.

[21] Legro RS, Arslanian SA, Ehrmann DA (2013). Diagnosis and treatment of polycystic ovary syndrome: an Endocrine Society clinical practice guideline. J Clin Endocrinol Metab.

[22] Teede HJ, Misso ML, Costello MF (2018). Recommendations from the international evidence-based guideline for the assessment and management of polycystic ovary syndrome. Hum Reprod.

[23] Joo YY, Actkins K, Pacheco JA, et al. A polygenic and phenotypic risk prediction for polycystic ovary syndrome evaluated by phenome-wide association studies. J Clin Endocrinol Metab. 2020;105(6) 10.1210/clinem/dgz326. 10.1210/clinem/dgz326 PMC7453038 31917831

[24] Pedersen SD, Brar S, Faris P, Corenblum B (2007). Polycystic ovary syndrome: validated questionnaire for use in diagnosis. Can Fam Physician.

[25] Zhang XZ, Pang YL, Wang X, Li YH (2018). Computational characterization and identification of human polycystic ovary syndrome genes. Sci Rep.

[26] Deshmukh H, Papageorgiou M, Kilpatrick ES, Atkin SL, Sathyapalan T (2019). Development of a novel risk prediction and risk stratification score for polycystic ovary syndrome. Clin Endocrinol.

[27] Sun Q, Yang Y, Peng X (2019). Coagulation parameters predictive of polycystic ovary syndrome. Eur J Obstet Gynecol Reprod Biol.

[28] Chen ZJ, Zhao H, He L (2011). Genome-wide association study identifies susceptibility loci for polycystic ovary syndrome on chromosome 2p16.3, 2p21 and 9q33.3. Nat Genet.

[29] Shi Y, Zhao H, Shi Y (2012). Genome-wide association study identifies eight new risk loci for polycystic ovary syndrome. Nat Genet.

[30] Goodarzi MO, Jones MR, Li X (2012). Replication of association of DENND1A and THADA variants with polycystic ovary syndrome in European cohorts. J Med Genet.

[31] Welt CK, Styrkarsdottir U, Ehrmann DA (2012). Variants in DENND1A are associated with polycystic ovary syndrome in women of European ancestry. J Clin Endocrinol Metab.

[32] Louwers YV, Stolk L, Uitterlinden AG, Laven JS (2013). Cross-ethnic meta-analysis of genetic variants for polycystic ovary syndrome. J Clin Endocrinol Metab.

[33] Brower MA, Jones MR, Rotter JI (2015). Further investigation in europeans of susceptibility variants for polycystic ovary syndrome discovered in genome-wide association studies of Chinese individuals. J Clin Endocrinol Metab.

[34] Day F, Karaderi T, Jones MR (2018). Large-scale genome-wide meta-analysis of polycystic ovary syndrome suggests shared genetic architecture for different diagnosis criteria. PLoS Genet.

[35] Vagios S, James KE, Sacha CR (2021). A patient-specific model combining antimullerian hormone and body mass index as a predictor of polycystic ovary syndrome and other oligo-anovulation disorders. Fertil Steril.

[36] Strowitzki T (2021). Advanced diagnosis of polycystic ovary syndrome-new prediction models with standard parameters. Fertil Steril.

[37] Bouzas IC, Cader SA, Leao L, Kuschnir MC, Braga C (2014). Menstrual cycle alterations during adolescence: early expression of metabolic syndrome and polycystic ovary syndrome. J Pediatr Adolesc Gynecol.

[38] Li M, Ruan X, Ju R (2022). Is anti-Mullerian hormone a useful biomarker in the diagnosis of polycystic ovary syndrome in Chinese adolescents?. Gynecol Endocrinol.

[39] Zigarelli A, Jia Z, Lee H (2022). Machine-aided self-diagnostic prediction models for polycystic ovary syndrome: observational study. JMIR Form Res.

[40] Bahri Khomami M, Joham AE, Boyle JA (2019). Increased maternal pregnancy complications in polycystic ovary syndrome appear to be independent of obesity-a systematic review, meta-analysis, and meta-regression. Obes Rev.

[41] Xia H, Zhang R, Sun X, Wang L, Zhang W (2017). Risk factors for preeclampsia in infertile Chinese women with polycystic ovary syndrome: a prospective cohort study. J Clin Hypertens.

[42] Lewandowski KC, Skowronska-Jozwiak E, Lukasiak K (2019). How much insulin resistance in polycystic ovary syndrome? Comparison of HOMA-IR and insulin resistance [Belfiore] index models. Arch Med Sci.

[43] Jiang F, Wei K, Lyu W, Wu C (2020). Predicting risk of insulin resistance in a Chinese population with polycystic ovary syndrome: designing and testing a new predictive nomogram. Biomed Res Int.

[44] Cibula D, Skrha J, Hill M (2002). Prediction of insulin sensitivity in nonobese women with polycystic ovary syndrome. J Clin Endocrinol Metab.

[45] Gennarelli G, Holte J, Berglund L, Berne C, Massobrio M, Lithell H (2000). Prediction models for insulin resistance in the polycystic ovary syndrome. Hum Reprod.

[46] Bahri Khomami M, Earnest A, Loxton D, Teede HJ, Joham AE (2021). Predictors of hypertensive disorders in pregnancy in women with and without polycystic ovary syndrome: the Australian longitudinal study of Women's health. Clin Endocrinol.

[47] Pan H, Xian P, Yang D (2021). Polycystic ovary syndrome is an independent risk factor for hypertensive disorders of pregnancy: a systematic review, meta-analysis, and meta-regression. Endocrine.

[48] Mills G, Badeghiesh A, Suarthana E, Baghlaf H, Dahan MH (2020). Polycystic ovary syndrome as an independent risk factor for gestational diabetes and hypertensive disorders of pregnancy: a population-based study on 9.1 million pregnancies. Hum Reprod.

[49] Boomsma CM, Eijkemans MJ, Hughes EG, Visser GH, Fauser BC, Macklon NS (2006). A meta-analysis of pregnancy outcomes in women with polycystic ovary syndrome. Hum Reprod Update.

[50] de Wilde MA, Veltman-Verhulst SM, Goverde AJ (2014). Preconception predictors of gestational diabetes: a multicentre prospective cohort study on the predominant complication of pregnancy in polycystic ovary syndrome. Hum Reprod.

[51] Wang J, Lv B, Chen X (2021). An early model to predict the risk of gestational diabetes mellitus in the absence of blood examination indexes: application in primary health care centres. BMC Pregnancy Childbirth.

[52] Naver KV, Grinsted J, Larsen SO (2014). Increased risk of preterm delivery and pre-eclampsia in women with polycystic ovary syndrome and hyperandrogenaemia. BJOG.

[53] Palomba S, Falbo A, Russo T, Tolino A, Orio F, Zullo F (2010). Pregnancy in women with polycystic ovary syndrome: the effect of different phenotypes and features on obstetric and neonatal outcomes. Fertil Steril.

[54] de Wilde MA, Lamain-de Ruiter M, Veltman-Verhulst SM (2017). Increased rates of complications in singleton pregnancies of women previously diagnosed with polycystic ovary syndrome predominantly in the hyperandrogenic phenotype. Fertil Steril.

[55] Palomba S, de Wilde MA, Falbo A, Koster MP, La Sala GB, Fauser BC (2015). Pregnancy complications in women with polycystic ovary syndrome. Hum Reprod Update.

[56] Christ JP, Gunning MN, Meun C (2019). Pre-conception characteristics predict obstetrical and neonatal outcomes in women with polycystic ovary syndrome. J Clin Endocrinol Metab.

[57] Bazarganipour F, Ziaei S, Montazeri A, Foroozanfard F, Kazemnejad A, Faghihzadeh S (2014). Health-related quality of life in patients with polycystic ovary syndrome [PCOS]: a model-based study of predictive factors. J Sex Med.

[58] Bazarganipour F, Ziaei S, Montazeri A, Foroozanfard F, Kazemnejad A, Faghihzadeh S (2013). Predictive factors of health-related quality of life in patients with polycystic ovary syndrome: a structural equation modeling approach. Fertil Steril.

[59] Aboulghar MA, Mansour RT (2003). Ovarian hyperstimulation syndrome: classifications and critical analysis of preventive measures. Hum Reprod Update.

[60] Fauser BC, Devroey P, Macklon NS (2005). Multiple birth resulting from ovarian stimulation for subfertility treatment. Lancet.

[61] Ovarian Stimulation T, Bosch E, Broer S (2020). ESHRE guideline: ovarian stimulation for IVF/ICSI[dagger]. Hum Reprod Open.

[62] Li F, Chen Y, Niu A, He Y, Yan Y (2021). Nomogram model to predict the probability of Ovarian Hyperstimulation syndrome in the treatment of patients with polycystic ovary syndrome. Front Endocrinol.

[63] Cao Y, Shi H, Ma Y, Ma L, Zhai J (2020). Effect and relationship of seasons on the high risk of Ovarian Hyperstimulation syndrome after oocyte retrieval in patients with polycystic ovary syndrome. Front Endocrinol.

[64] Guzman L, Ortega-Hrepich C, Polyzos NP (2013). A prediction model to select PCOS patients suitable for IVM treatment based on anti-Mullerian hormone and antral follicle count. Hum Reprod.

[65] Imani B, Eijkemans MJ, te Velde ER, Habbema JD, Fauser BC (1998). Predictors of patients remaining anovulatory during clomiphene citrate induction of ovulation in normogonadotropic oligoamenorrheic infertility. J Clin Endocrinol Metab.

[66] Imani B, Eijkemans MJ, te Velde ER, Habbema JD, Fauser BC (1999). Predictors of chances to conceive in ovulatory patients during clomiphene citrate induction of ovulation in normogonadotropic oligoamenorrheic infertility. J Clin Endocrinol Metab.

[67] Imani B, Eijkemans MJ, te Velde ER, Habbema JD, Fauser BC (2002). A nomogram to predict the probability of live birth after clomiphene citrate induction of ovulation in normogonadotropic oligoamenorrheic infertility. Fertil Steril.

[68] Eijkemans MJ, Imani B, Mulders AG, Habbema JD, Fauser BC (2003). High singleton live birth rate following classical ovulation induction in normogonadotrophic anovulatory infertility [WHO 2]. Hum Reprod.

[69] van Wely M, Bayram N, van der Veen F, Bossuyt PM (2005). Predicting ongoing pregnancy following ovulation induction with recombinant FSH in women with polycystic ovary syndrome. Hum Reprod.

[70] Rausch ME, Legro RS, Barnhart HX (2009). Predictors of pregnancy in women with polycystic ovary syndrome. J Clin Endocrinol Metab.

[71] Nyboe Andersen A, Balen AH, Platteau P, Pettersson G, Arce JC (2010). Prestimulation parameters predicting live birth in anovulatory WHO Group II patients undergoing ovulation induction with gonadotrophins. Hum Reprod.

[72] van Santbrink EJ, Hop WC, Fauser BC (1997). Classification of normogonadotropic infertility: polycystic ovaries diagnosed by ultrasound versus endocrine characteristics of polycystic ovary syndrome. Fertil Steril.

[73] Broekmans FJ, Knauff EA, Valkenburg O, Laven JS, Eijkemans MJ, Fauser BC (2006). PCOS according to the Rotterdam consensus criteria: change in prevalence among WHO-II anovulation and association with metabolic factors. BJOG.

[74] Verit FF, Erel O, Kocyigit A (2007). Association of increased total antioxidant capacity and anovulation in nonobese infertile patients with clomiphene citrate-resistant polycystic ovary syndrome. Fertil Steril.

[75] van Wely M, Fauser BC, Laven JS, Eijkemans MJ, van der Veen F (2006). Validation of a prediction model for the follicle-stimulating hormone response dose in women with polycystic ovary syndrome. Fertil Steril.

[76] Jin H, Shen X, Song W, Liu Y, Qi L, Zhang F (2021). The development of nomograms to predict Blastulation rate following cycles of in vitro fertilization in patients with tubal factor infertility, polycystic ovary syndrome, or endometriosis. Front Endocrinol.

[77] Legro RS, Brzyski RG, Diamond MP (2014). The pregnancy in polycystic ovary syndrome II study: baseline characteristics and effects of obesity from a multicenter randomized clinical trial. Fertil Steril.

[78] Kuang H, Jin S, Hansen KR (2015). Identification and replication of prediction models for ovulation, pregnancy and live birth in infertile women with polycystic ovary syndrome. Hum Reprod.

[79] Gao L, Li M, Wang Y (2020). Overweight and high serum total cholesterol were risk factors for the outcome of IVF/ICSI cycles in PCOS patients and a PCOS-specific predictive model of live birth rate was established. J Endocrinol Investig.

[80] Jiang X, Liu R, Liao T (2021). A predictive model of live birth based on obesity and metabolic parameters in patients with PCOS undergoing frozen-thawed embryo transfer. Front Endocrinol.

[81] Veltman-Verhulst SM, Fauser BC, Eijkemans MJ (2012). High singleton live birth rate confirmed after ovulation induction in women with anovulatory polycystic ovary syndrome: validation of a prediction model for clinical practice. Fertil Steril.

[82] Guan HJ, Pan LQ, Song H, Tang HY, Tang LS (2021). Predictors of pregnancy after intrauterine insemination in women with polycystic ovary syndrome. J Int Med Res.

[83] Escobar-Morreale HF (2018). Polycystic ovary syndrome: definition, aetiology, diagnosis and treatment. Nat Rev Endocrinol.

[84] Sadeghi HM, Adeli I, Calina D, et al. Polycystic ovary syndrome: a comprehensive review of pathogenesis, management, and drug repurposing. Int J Mol Sci. 2022;23(2) 10.3390/ijms23020583. 10.3390/ijms23020583 PMC8775814 35054768

[85] Eiras MC, Pinheiro DP, Romcy KAM, Ferriani RA, Reis RMD, Furtado CLM (2022). Polycystic ovary syndrome: the epigenetics behind the disease. Reprod Sci.

[86] Wolf WM, Wattick RA, Kinkade ON, Olfert MD. Geographical prevalence of polycystic ovary syndrome as determined by region and race/ethnicity. Int J Environ Res Public Health. 2018;15(11) 10.3390/ijerph15112589. 10.3390/ijerph15112589 PMC6266413 30463276

[87] Spremovic Radenovic S, Pupovac M, Andjic M, et al. Prevalence, Risk Factors, and Pathophysiology of Nonalcoholic Fatty Liver Disease [NAFLD] in Women with Polycystic Ovary Syndrome [PCOS]. Biomedicines. 2022;10(1) 10.3390/biomedicines10010131. 10.3390/biomedicines10010131 PMC8773533 35052811

[88] Falzarano C, Lofton T, Osei-Ntansah A (2022). Nonalcoholic fatty liver disease in women and girls with polycystic ovary syndrome. J Clin Endocrinol Metab.

[89] Zhou X, Jaswa E, Pasch L, Shinkai K, Cedars MI, Huddleston HG (2021). Association of obstructive sleep apnea risk with depression and anxiety symptoms in women with polycystic ovary syndrome. J Clin Sleep Med.

[90] Kahal H, Kyrou I, Uthman OA (2020). The prevalence of obstructive sleep apnoea in women with polycystic ovary syndrome: a systematic review and meta-analysis. Sleep Breath.

[91] Bayona A, Martinez-Vaello V, Zamora J, Nattero-Chavez L, Luque-Ramirez M, Escobar-Morreale HF. Prevalence of PCOS and related hyperandrogenic traits in premenopausal women with type 1 diabetes: a systematic review and meta-analysis. Hum Reprod Update. 2022; 10.1093/humupd/dmac011. 10.1093/humupd/dmac011 35237802

[92] Hudnut-Beumler J, Kaar JL, Taylor A (2021). Development of type 2 diabetes in adolescent girls with polycystic ovary syndrome and obesity. Pediatr Diabetes.

[93] Wan P, Meng L, Huang C (2021). Replication study and meta-analysis of selected genetic variants and polycystic ovary syndrome susceptibility in Asian population. J Assist Reprod Genet.

[94] Chaudhary H, Patel J, Jain NK, Joshi R (2021). The role of polymorphism in various potential genes on polycystic ovary syndrome susceptibility and pathogenesis. J Ovarian Res.

[95] Mir R, Saeedi NH, Jalal MM, et al. Clinical implications of Krupple-like transcription factor KLF-14 and certain Micro-RNA [miR-27a, miR-196a2, miR-423] gene variations as a risk factor in the genetic predisposition to PCOS. J Pers Med. 2022:12[4]. 10.3390/jpm12040586. 10.3390/jpm12040586 PMC9030665 35455702

[96] Zhang Z, Liu Y, Lv J (2021). Differential Lipidomic characteristics of children born to women with polycystic ovary syndrome. Front Endocrinol.

[97] Daan NM, Koster MP, Steegers-Theunissen RP, Eijkemans MJ, Fauser BC (2017). Endocrine and cardiometabolic cord blood characteristics of offspring born to mothers with and without polycystic ovary syndrome. Fertil Steril.

[98] Gunning MN, Sir Petermann T, Crisosto N (2020). Cardiometabolic health in offspring of women with PCOS compared to healthy controls: a systematic review and individual participant data meta-analysis. Hum Reprod Update.

[99] Xue Z, Li J, Feng J (2021). Research Progress on the mechanism between polycystic ovary syndrome and abnormal endometrium. Front Physiol.

[100] Zhao J, Chen Q, Xue X. An update on the Progress of endometrial receptivity in women with polycystic ovary syndrome. Reprod Sci. 2021; 10.1007/s43032-021-00641-z. 10.1007/s43032-021-00641-z 34076874

[101] Jiang NX, Li XL. The disorders of endometrial receptivity in PCOS and its mechanisms. Reprod Sci. 2021; 10.1007/s43032-021-00629-9. 10.1007/s43032-021-00629-9 34046867

[102] Leushuis E, van der Steeg JW, Steures P (2009). Prediction models in reproductive medicine: a critical appraisal. Hum Reprod Update.

[103] Justice AC, Covinsky KE, Berlin JA (1999). Assessing the generalizability of prognostic information. Ann Intern Med.

