# Supplementary material for: Progress of the application clinical prediction model in polycystic ovary syndrome
Source: J Ovarian Res. 2023 Nov 25;16:230. doi: 10.1186/s13048-023-01310-2 (PMC10675861; doi:10.1186/s13048-023-01310-2)
Supplement: Supplementary file 1 — Additional file 1: Supplement Material 1. PCOS diagnosis model. [file 13048_2023_1310_MOESM1_ESM.docx]

| **Supplementary Material 1. PCOS diagnosis model** | | | | | | | | |
| --- | --- | --- | --- | --- | --- | --- | --- | --- |
| **PCOS diagnosis model** | **The first author** | **year** | **country** | **methods** | **Variables/predictors** | **clinical application/ the validity** | **advantages and disadvantages** | **reference** |
| PCOS Polygenic Risk Score | Joo YY | 2020 | America | Phenome-wide association studies;  the electronic health records (EHR) of 124,852 individuals | combining polygenic risk scores (PRS)  with PCOS component phenotypes into a polygenic and phenotypic risk score (PPRS). | expand the methodological utility of PRS in PCOS patient; provide a novel methodological opportunity to stratify patients’ genetic risk and discover the network associated with PCOS pathogenesis. | improving cost-effectiveness for PCOS; detection of PCOS patients  prior to diagnosis; Improving understanding based on specific phenotypic or genetic variations. small sample size; polygenic prediction is limited to three representative phenotypes; selection bias; the low diagnosis rate of PCOS patients in  current EHR system. | (23) |
| PCOS | Pedersen SD | 2007 | Canada | questionnaire is used in diagnosis of PCOS  50 PCOS and  50 patients without PCOS | Mainly variable long  menstrual cycles(≥35 d); coarse hair at ≥  3 sites; history of obesity; lactation unrelated to pregnancy. Score value≥2, PCOS. | a simple clinical tool to help diagnose PCOS and it will be useful for family physicians’ busy practices. | It was validated using a second sample of 117 patients, in addition to internal validation.  The questionnaire has not been validated in a family medicine setting; sample size was relatively limited; | (24) |
| Predict PCOS genes | Zhang XZ | 2018 | China | supervised machine-learning algorithm;  306 PCOS genes as positive samples; 306 negative samples from 13,681 genes | 1001 times of the negative datasets of 306 genes | With a posterior probability higher than 0.9 233 new PCOS genes were predicted. Literature supporting 7 of the top 10 predictions has been found. | Different from GWAS and other genetic methods, it opens a new avenue to infer PCOS candidates; a number of false positive interactions and false negatives. | (25) |
| PCOS | Deshmukh H | 2019 | Britain | a simple phenotypic algorithm;  111 PCOS women  67 control women | Mainly FAI, AMH, WC, 17-OHP | assist in diagnosis, severity prediction of the disease risk stratification of PCOS women. | a simple 4-variable model can capture the underlying clinical and Hormonal abnormalities; lacking of externally validated; sample size was modest; all the patients had classical PCOS, not able to evaluate model for the other 3 phenotypes. | (26) |
| PCOS | Sun Q | 2019 | China | Coagulation parameters  181 PCOS patients and 301 health controls | Mainly six coagulation parameters; age | PT, TT, and FDP are predictive of PCOS; highlight the potential of anti-coagulation therapies. | Predict PCOS based on six coagulation parameters;  sample size was relatively limited; residual confounding  and imbalances between the PCOS and the control groups. | (27) |
| PCOS and other  oligo-anovulation disorders | Vagios S | 2021 | America | 228 PCOS patients  compared with BMI  and AMH values in 93 patients with ovulation disorders unrelated to PCOS and 689 patients with nonendocrine infertility | Mainly BMI and AMH | provide a prediction model in direct comparison to other forms of ovulatory disorders and to patients without  endocrine disorders. | support a definite diagnosis in patients with suspected PCOS, but it is  still far from allowing a valid discrimination from other forms  of ovulatory dysfunction; Besides, the group of patients with other ovulation disorders was small. | (35) |
| Adolescents PCOS | Li M | 2022 | China | 45PCOS and 45 control group  PCOS diagnostic criteria^1^ (the Endocrinology Expert Group, Obstetrics/Gynecology Branch of the Chinese Medical Association) | Mainly AMH and Total Testosterone | AMH and Total Testosterone are useful biomarker for the diagnosis of PCOS in Chinese adolescent girls. (AMH cut off value of 6.32 ng/mL). | study focused on adolescent girls with PCOS; a relatively small sample size; different AMH assays, different diagnostic criteria  for PCOS and genetical and other differences in the patient populations. | (38) |
| Machine-Aided Self-diagnostic PCOS | Zigarelli A | 2022 | America | machine learning techniques  PCOS-positive size (n=170)  PCOS-negative size (n=356) | noninvasive variables; All variables including  noninvasive and invasive variables. | models serve as a convenient digital platform;  help diagnose PCOS | two kinds of models;  users can acquire pre- or self-diagnosis and counsel for the risk of PCOS, with or without obtaining medical test results;  access the platform at home without delay. need further externally validated. | (39) |

^1^Endocrinology group and guidelines expert group of the Chinese medical association obstetrics and gynecology branch. Guidelines for diagnosis and treatment of polycystic ovary syndrome in China. Chin J Obstet Gynecol. 2018; 53:2–6.

PT: Prothrombin time; TT: Thrombin time; FDP: Fibrin degradation products; BMI: Body mass index;

FAI: free androgen index; AMH: anti-Müllerian hormone; WC: waist circumference; 17OHP: 17α-hydroxyprogesterone; GWAS: Genome-Wide Association Studies.
