# Supplementary material for: Progress of the application clinical prediction model in polycystic ovary syndrome
Source: J Ovarian Res. 2023 Nov 25;16:230. doi: 10.1186/s13048-023-01310-2 (PMC10675861; doi:10.1186/s13048-023-01310-2)
Supplement: Supplementary file 2 — Additional file 2: Supplement Material 2. Prediction model of PCOS complications. [file 13048_2023_1310_MOESM2_ESM.docx]

| **Supplementary Material 2. Prediction model of PCOS complications** | | | | | | | | |
| --- | --- | --- | --- | --- | --- | --- | --- | --- |
| **Prediction model of PCOS complications** | **The first author** | **year** | **country** | **methods** | **Variables/ predictors** | **clinical application/ the validity** | **advantages and disadvantages** | **reference** |
| IR risk prediction tools | Jiang F | 2020 | China | nomogram;1000 bootstrap resampling; the LASSO binary logistic regression model;  101 IR PCOS and 44 PCOS without IR | Mainly BMI, employment, the duration of disease, whether they take metformin at present, and the activity time. | provide a predictive method for IR in PCOS  patients; screen out  the high-risk population of IR; gain a time window for clinical intervention and treatment. | The first nomogram  studied PCOS and resistance to insulin;  collected data represents some PCOS women; lacking of evaluated externally; model relatively accurate. | (43) |
| HDP | Bahri Khomami M | 2021 | Australian | analysis of the Australian Longitudinal Study on Women's Health (ALSWH); 14,247 participants; 5838 (492 PCOS)  participants with 34,182pregnancies over 19 years | Mainly age, BMI, country of birth,  parity, multiple pregnancy, subfertility, infertility treatment, GDM, family history of GDM and socioeconomic  status. | confirm that management of GDM may compensate the risk of HDP in women with PCOS. | the first study to investigate HDP and  associated risk factors in relation to PCOS status; recall bias; PCOS diagnosis and HDP were self- reported; results may only pertain to women born 1973– 1978. | (46) |
| GDM (PCOS preconception prediction model) | de Wilde MA | 2014 | Netherlands | a multi-center prospective cohort study; 326 PCOS, 189 pregnancies PCOS;148 pregnancies PCOS without GDM; 41 with GDM. | first-degree relatives with type 2 diabetes mellitus, serum levels of fasting glucose, fasting insulin, androstenedione and SHBG  before conception | identify PCOS women particularly at risk for GDM in preconception. | the first PCOS preconception prediction GDM model; Lacking of externally validated; existing missing data; a relatively small sample size. | (50) |
| GDM (without blood examination indexes) | Wang J | 2021 | China | machine learning algorithm (Random Forest model and Logistic Regression model); 1139 PCOS pregnant women (186 with GDM) | pre-pregnancy BMI, AC in the first trimester, age, PCOS, gravidity, irregular menstruation, and family history of diabetes. | early predictive model of GDM for  the primary health care center based on several simple  variables without blood examination indexes. | not increase the  psychological and economic burden, suitable for primary health care centers. simple and inexpensive but practical; a single region data; lacking of external verification; a relatively small sample size. | (51) |
| Obstetric and neonatal outcomes (based on pre-conception characteristics) | Christ JP | 2018 | Netherlands | two large data  resources of 2768 PCOS before conception and the Dutch Perinatal national registry (including 1715 pregnancies and 1786 offspring) | Hyperandrogenism; impaired glucose tolerance; androgen  Levels. | PCOS preconception hyperandrogenism and impaired glucose  tolerance predict multiple obstetric and perinatal complications; androgen levels independently predicted development of preeclampsia, preterm deliveries, and any adverse obstetric  or perinatal outcome. | data analysis large;  not lost to follow-up by national database; Recruitment was primarily of infertile women  presenting with menstrual cycle irregularities and few  women with regular cycles were included;  data (paternal features, method of conception, or monitoring and interventions during pregnancy) was limited; lacking of external verification. | (56) |
| Health-Related Quality of Life in PCOS patients | Bazarganipour F | 2014 | Iran | a cross-sectional study of 300 women with PCOS; structural equation modeling (SEM) approach | questionnaires: the Hospital Anxiety  and Depression Scale, the Body Image Concern Inventory, the Rosenberg’s Self-Esteem Scale score, the modified PCOS health-related quality of life questionnaire, the Female Sexual Function Index. | pay attention to PCOS Health-Related Quality of Life., especially self-esteem, body image, and sexual function and adequately treated for PCOS patients. | point out the mediating factors for PCOS patients about Health-Related Quality of Life;  data were self-reported; cross-sectional study, difficult to determine the causality; source of the patient; need to be confirmed in a longitudinal study | (57) |

IR: insulin resistance; HDP: hypertensive disorders of pregnancy; GDM: gestational diabetes mellitus; LASSO: Least absolute shrinkage and selection operator; SHBG: sex hormone-binding globulin; AC: abdomen circumference;
