# Supplementary material for: Progress of the application clinical prediction model in polycystic ovary syndrome
Source: J Ovarian Res. 2023 Nov 25;16:230. doi: 10.1186/s13048-023-01310-2 (PMC10675861; doi:10.1186/s13048-023-01310-2)
Supplement: Supplementary file 3 — Additional file 3: Supplement Material 3. Prediction model of PCOS treatment outcomes. [file 13048_2023_1310_MOESM3_ESM.docx]

| **Supplementary Material 3. Prediction model of PCOS treatment outcomes** | | | | | | | | |
| --- | --- | --- | --- | --- | --- | --- | --- | --- |
| **Prediction model of PCOS treatment outcomes** | **The first author** | **year** | **country** | **methods** | **Variables/ predictors** | **clinical application/ the validity** | **advantages and disadvantages** | **reference** |
| OHSS | Li F | 2021 | China | Nomogram;  4351 PCOS patients (modeling group 3231 cases; verification group 1120 cases);  986 OHSS; 3365 without OHSS. | FSH, AMH, E2  values and follicle number on the day of HCG injection, total dosage of Gn used. | a useful tool in helping physicians and the PCOS patients, to decide on a treatment option before IVF/ICSI; the clinician can set up a better clinical management strategy for  conducting a precise personal therapy. | effectively, easily, and intuitively predict the probability of OHSS in PCOS patients; retrospective study (potential biases); a certain deficiency in the universality and extrapolation of research. | (62) |
| OHSS | Cao Y | 2021 | China | Nomogram;  2030 PCOS patients  (683 high risk of OHSS and 1347 controls) | Mainly total Gn dosage, number of  retrieved oocytes, E2 level, and average diameter of the bilateral  ovaries on the HCG injection day. | Clinicians can conduct personalized and effective prevention and treatment  measures for PCOS patients to reduce the risk of OHSS. | nomogram studied the incidence of PCOS patients at high risk for OHSS after oocyte retrieval in the cycle of IVF/ICSI; risk fluctuated with temperature changes; limited by region and sample size; need further externally validated. | (63) |
| PCOS patients who are suitable for IVM treatment | Guzman L | 2013 | Belgium | 124 consecutive patients with  PCOS were recruited for IVM treatment. | BMI, baseline AMH, FSH, E2, testosterone and AFC | help ART practitioners to identify patients  who are suitable for (IVM) treatment | patient selection tool to predict the probability to obtain at least eight COC in a non-HCG-triggered IVM cycle in PCOS patients; oocyte yield in a IVM system does not only depend on patient parameters; lack of a standardized IVM system; the heterogeneity of the patient, it needs a second independent validation sample. | (64) |
| PCOS ongoing pregnancy model | van Wely M | 2005 | Netherlands | 85 women with CC-resistant PCOS; logistic regression analyses. | Clinical, ultrasonographic endocrinological parameters. | Clinicians and patients can predict the possibility of ongoing pregnancy after ovulation  induction. | prediction chance of achieving an ongoing pregnancy after ovulation induction with rFSH in women with CC-resistant PCOS; model discriminative  power was modest;  included only women with CC-resistant PCOS; sample size small; lacking of external validation. | (69) |
| The response to ovulation induction | Verit FF | 2007 | Turkey | Prospective longitudinal follow-up study; 55 nonobese, oligomenorrheic women with PCOS and normal indices of  insulin sensitivity (28 CC responder; 27 CC resistant). | Mainly TAC, FAI, and OV. | Clinicians and patients can predict the anovulatory possibility after CC medication based on TAC, FAI, and OV. | predict the anovulatory possibility by simple parameters; deficiency in the universality and extrapolation of research; sample size small; lacking of validation sample. | (74) |
| The individual FSH response dose | van Wely M | 2006 | Netherlands | 85CC–resistant PCOS patients；  based on 90 PCOS patients who were unsuccessful treatment with CC (constructed published model). | BMI, CC response, initial serum FSH level, and initial  serum insulin-to-glucose ratio | It proved that a published model performance was poor. | validate a published model for the prediction of the individual FSH response dose for Gn  induction of ovulation in PCOS; sample size small; find that predictive performance of the model was poor. | (75) |
| Blastocyst formation rate following cycles of IVF | Jin H | 2021 | China | Nomogram;  1691 PCOS  (Training cohort:1128; Validation cohort:491) | Age, AMH, the number of oocytes retrieve, fertilization rate, the rate of  top-quality embryos on day 3. | clinicians can obtain a total score to guide the management  of patients with regard to extended culture to the blastocyst stage.; patients can be told the probability of blastocyst formation. | a simple tool which guides the management about extended culture to the blastocyst stage;  lacking of external validation; embryos that were  extended culture to the blastocyst stage were the remaining  embryos that showed normal fertilization after fresh transfer  and freezing on day 3; only predicted the average blastocyst formation rate for the  entire embryo group; retrospective study (potential bias). | (76) |
| PCOS models of ovulation,  pregnancy and live birth | Kuang H | 2015 | America | secondary analysis of the data from the pregnancy in PCOS I and II (PPCOS-I and -II) trials; validation models and constructed new prediction models | Mainly Age, FAI, insulin, the time of conception, and SHBG | prediction ovulation and pregnancy outcomes in infertile women with PCOS based on a simple predictive model | first evaluated  previously reported significant predictors, and then constructed new prediction models.  large sample size; models require further validation. | (78) |
| PCOS-specific predictive model of live birth rate | Gao L | 2020 | China | Nomogram;  259 PCOS patients;  modeling group:178  verification group:81 | Mainly BMI, TC, basal FSH, type of embryo transferred and age. | helping physicians and PCOS women decide on a treatment option and pre-processing before IVF/ICSI. | the first to predict the individual probability of a live birth for women with PCOS-related infertility; retrospective study (potential biases); lacking of external validation; many other vital factors were not taken into account. | (79) |
| Live birth based on obesity and metabolic parameters | Jiang X | 2021 | China | Nomogram;  1158 PCOS patients that were clinically pregnant following  F-ET treatment； modeling group:928  Verification group:230 | Mainly advanced age, obesity, serum TC, TG, and IR | predict the odds of live birth for PCOS patients undergoing F-ET, and improve the efficiency of pre-transfer management. | The first study predicts live birth rates for PCOS patients following F-ET； sample size was relatively large； potential risk factors were considered； lacking of external validation. | (80) |
| Singleton live birth rate | Veltman-Verhulst SM | 2012 | Netherlands | Validation cohort of 108 treatment-naïve anovulatory PCOS patients;  prospective follow-up study (73 singleton live birth, 35 no singleton live birth) | Mainly age, duration of infertility, BMI,  hyperandrogenism, IR, and insulin-to-glucose ratio. | this model has good clinical potential,  patients with a low predicted live birth chance can now easily be identified. | Validation of the initial model (Variables: age, duration of infertility, and insulin-to-glucose ratio)；and validation of alternative model (Variables: age,  duration of infertility, and BMI); discriminating between couples with a poor prognosis or a good prognosis. (a poor individual discriminative performance); small sample size; need further validation. | (81) |
| Predictors of pregnancy after IUI | Guan HJ | 2021 | China | retrospective study;  831 IUI cycles in 451 couples with PCOS;  (188 pregnant; 643 without pregnant) | Mainly BMI, treatment cycles, treatment schemes, number of dominant follicles, endometrial thickness, infertility duration and type of  infertility. | obese PCOS women might require more Gn doses and more days of COS to overcome the effects of weight. guide doctor better management for obese PCOS patients. | evaluate the effects of BMI in patients with PCOS undergoing COS with IUI.; the small sample size;  retrospective study design (recall bias); need further validation. | (82) |

OHSS: ovarian hyperstimulation syndrome; FSH: Follicle-Stimulating Hormone; E2: estradiol; AMH: Anti-Mullerian hormone; HCG: Human Chorionic Gonadotropin; Gn: gonadotropins; IVM: In Vitro Maturation; AFC: Antral Follicle Counting; BMI: Body Mass Index; ART: assisted reproductive technology; COC: cumulus oocyte complexes; CC: clomiphene citrate; rFSH: recombinant human follicle stimulating hormone; TAC: total antioxidant capacity; OV: Ovarian volume; FAI: free androgen index; SHBG: sex hormone-binding globulin; IVF: in vitro fertilization; ICSI: intracytoplasmic sperm injection; TC: total serum cholesterol; F-ET: frozen-thawed embryo transfer; IUI: intrauterine insemination; COS: controlled ovarian stimulation
